# Overexpression of Larch *SCL6* Inhibits Transitions from Vegetative Meristem to Inflorescence and Flower Meristem in *Arabidopsis thaliana* (L.) Heynh.

**DOI:** 10.3390/plants13091232

**Published:** 2024-04-29

**Authors:** Jun-Xia Xing, Qiao-Lu Zang, Zha-Long Ye, Li-Wang Qi, Ling Yang, Wan-Feng Li

**Affiliations:** 1 State Key Laboratory of Tree Genetics and Breeding, School of Forestry, Northeast Forestry University, Harbin 150040, China; noyaxxx@163.com; 2 State Key Laboratory of Tree Genetics and Breeding, Key Laboratory of Tree Breeding and Cultivation of the National Forestry and Grassland Administration, Research Institute of Forestry, Chinese Academy of Forestry, Beijing 100091, China; zangql@sxau.edu.cn (Q.-L.Z.); kemiye@caf.ac.cn (Z.-L.Y.); lwqi@caf.ac.cn (L.-W.Q.); 3 College of Horticulture, Shanxi Agricultural University, Taigu 030801, China

**Keywords:** *HAM*, *Larix*, life cycle, longevity, miR171, time

## Abstract

*SCARECROW-LIKE6* (*SCL6*) plays a role in the formation and maintenance of the meristem. In *Larix kaempferi* (Lamb.) Carr., an important afforestation tree species in China, *SCL6* (*LaSCL6*) has two alternative splicing variants—*LaSCL6-var1* and *LaSCL6-var2*—which are regulated by microRNA171. However, their roles are still unclear. In this study, *LaSCL6-var1* and *LaSCL6-var2* were transformed into the *Arabidopsis thaliana* (L.) Heynh. genome, and the phenotypic characteristics of transgenic *A. thaliana*, including the germination percentage, root length, bolting time, flower and silique formation times, inflorescence axis length, and branch and silique numbers, were analyzed to reveal their functions. It was found that *LaSCL6-var1* and *LaSCL6-var2* overexpression shortened the root length by 41% and 31%, respectively, and increased the inflorescence axis length. Compared with the wild type, the bolting time in transgenic plants was delayed by approximately 2–3 days, the first flower and silique formation times were delayed by approximately 3–4 days, and the last flower and silique formation times were delayed by about 5 days. Overall, the life cycle in transgenic plants was prolonged by approximately 5 days. These results show that *LaSCL6* overexpression inhibited the transitions from the vegetative meristem to inflorescence meristem and from the flower meristem to meristem arrest in *A. thaliana,* revealing the roles of *LaSCL6-var1* and *LaSCL6-var2* in the fate transition and maintenance of the meristem.

## 1. Introduction

Meristem cells can divide continuously to produce new cells. The cells of the meristem form other types of tissue through division, growth, and differentiation, which are directly related to the growth and development of plants [1,2,3]. Many studies have shown that GRAS (GAI-RGA-SCR) transcription factors are involved in the growth and development of plants by regulating meristem activity [1,4,5,6]; for example, they control the indeterminacy and proliferation of shoot apical meristems and the de novo formation of axillary meristems [1,6,7,8,9].

*SCARECROW-LIKE6* (*SCL6*) belongs to the GRAS family [5,10]. The loss of function of *SCL6* in *Petunia hybrida* leads to the early termination of shoot apical meristems, arrested axillary shoot development, and a reduced number of carpels and stamens [11]. In addition, the *ham1 ham2 ham3* triple mutant leads to delayed inflorescence initiation, the early termination of shoot meristems, a disorganized meristem structure and morphology, and reduced axillary shoot branches [3,4,7,12]. These studies indicate that *SCL6* regulates meristem activity.

*SCL6* can be negatively regulated by microRNA171 (miR171) [12]. miR171 specifically recognizes and binds to the *SCL6* mRNA to mediate its cleavage [13,14]. The *miR171-SCL6* module participates in various developmental and physiological processes, including shoot branching [7,12,15,16], phase transition [1,17], root growth [12,18], inflorescence axis elongation [17,19,20,21], trichome initiation [22], silique production [20,21,23], and meristem development [9,18,24,25,26]. These findings indicate that the *miR171-SCL6* module participates in the activity and fate transition of the meristem.

*Larix kaempferi* (Lamb.) Carr. is an important coniferous timber tree species in China. *L. kaempferi SCL6* (*LaSCL6*) has two alternative splicing variants, *LaSCL6-var1* and *LaSCL6-var2*, both of which can be regulated by miR171 [27,28,29]. However, their roles are still unclear. The objective of this study is to explore their roles in life cycle progression based on the meristem state. We constructed overexpression vectors of *LaSCL6-var1* and *LaSCL6-var2* and transformed them into the *A. thaliana* genome; by analyzing the phenotypes of transgenic *A. thaliana* with respect to life cycle progression, their functions were explored. This study aimed to provide additional functional information on *SCL6*.

## 2. Results

### 2.1. Successful Transformation of LaSCL6 into A. thaliana Genome

**Figure 1:**
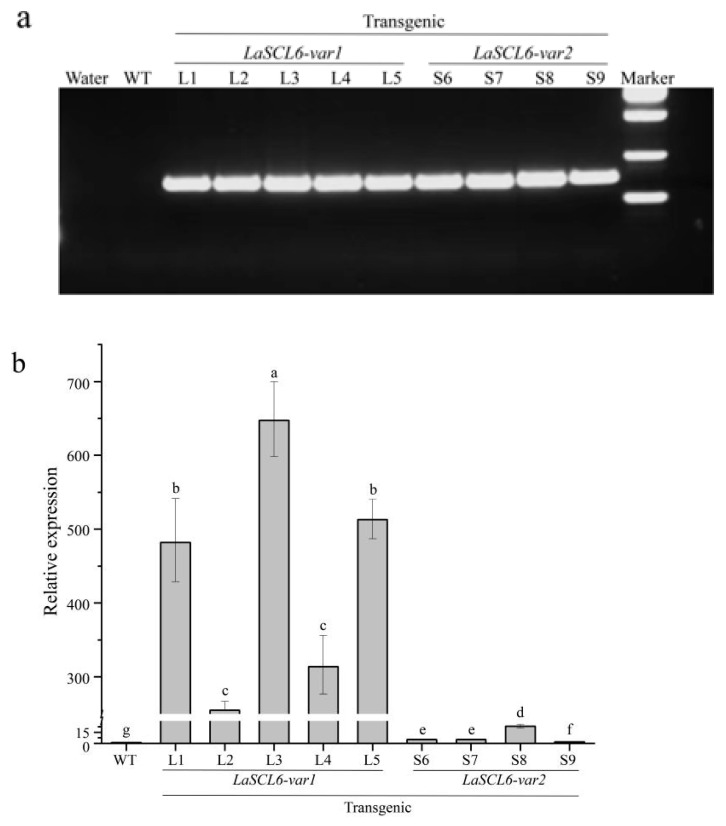
Verification of transgenic *Arabidopsis thaliana*. (**a**) PCR amplification of *LaSCL6* from wild-type (WT) and transgenic genomic DNA. (**b**) Relative expression levels of *LaSCL6* measured via qRT-PCR with *AtUBQ1* as internal control. Error bars represent standard deviations of three replicates. The differences between each line were analyzed using LSD, *p* ≤ 0.05, indicated by lowercase letters.

Five *LaSCL6-var1* (L1, L2, L3, L4, and L5) and four *LaSCL6-var2* (S6, S7, S8, and S9) overexpressing lines were randomly selected for the experiments. To verify the insertion of *LaSCL6* into the *A. thaliana* genome, polymerase chain reaction (PCR) amplification was performed with *LaSCL6*-specific primers and with the *A. thaliana* DNA as a template. The results showed that the amplified fragments of *LaSCL6* were detected in the transformed *A. thaliana* but not in the wild-type *A. thaliana* (Figure 1a). With the *A. thaliana* cDNA as a template, quantitative reverse transcription PCR (qRT-PCR) was performed to detect *LaSCL6* expression. The results showed that it was expressed in all transformed plants at different levels, but not in the wild-type *A. thaliana* (Figure 1b). These results indicate that *LaSCL6* was successfully integrated into the genome of *A. thaliana* and expressed.

### 2.2. LaSCL6 Overexpression Inhibits Root Elongation in A. thaliana

**Figure 2:**
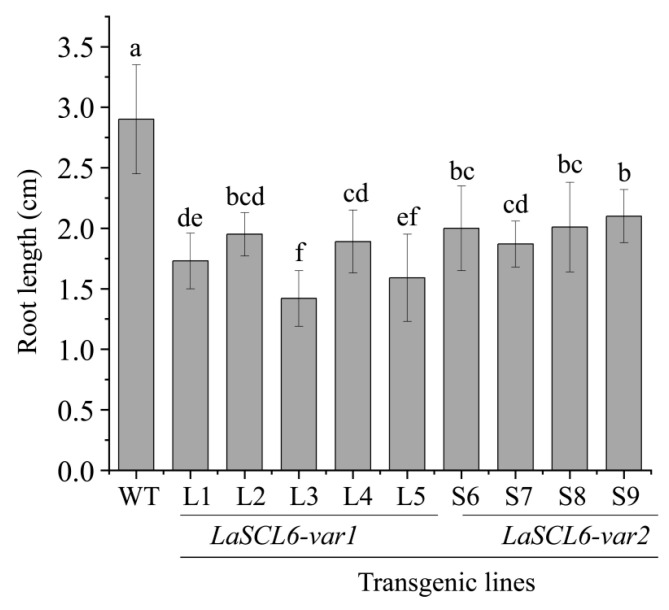
Root lengths of wild-type (WT) and transgenic *Arabidopsis thaliana*. The root length was measured after the plants were transferred into the incubator for 8 days. For each line, twenty plants were counted. The differences between each line were analyzed using LSD, *p* ≤ 0.05, indicated by lowercase letters.

After measuring the root length of *A. thaliana*, we found that *LaSCL6* overexpression inhibited root elongation because the root length in the transgenic *A. thaliana* was shorter than that in the wild-type *A. thaliana* (Figure 2). It was 2.90 cm (mean ± SD, 2.90 ± 0.45) in the wild-type *A. thaliana*, while it was 1.10–2.30 cm (mean ± SD, 1.71 ± 0.31; shortened by 41%) and 1.30–2.80 cm (mean ± SD, 1.99 ± 0.30; shortened by 31%) in the *LaSCL6-var1*- and *LaSCL6-var2*-overexpressing *A. thaliana*, respectively (Figure 2).

### 2.3. LaSCL6 Overexpression Has Almost No Influence on Reactivation of Dormant Meristem

**Figure 3:**
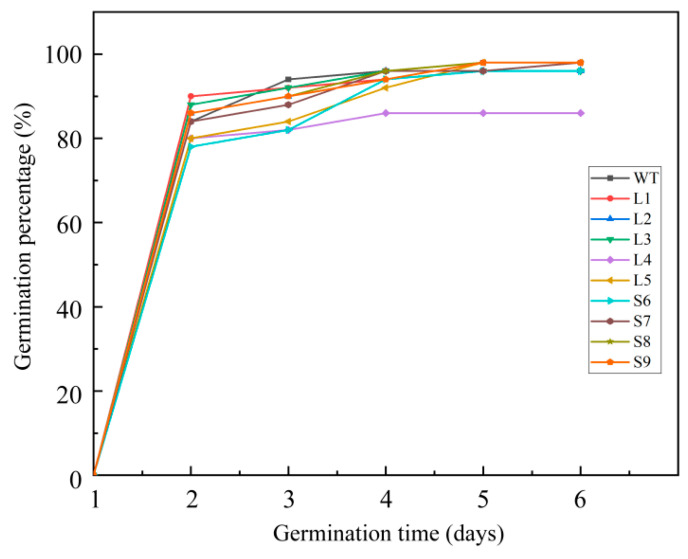
Germination percentages of wild-type (WT) and transgenic *Arabidopsis thaliana*. *LaSCL6-var1*-overexpressing *A. thaliana* lines: L1, L2, L3, L4, L5. *LaSCL6-var2*-overexpressing *A. thaliana* lines: S6, S7, S8, S9. Fifty seeds were used in each line.

After 4 °C treatment, 78–90% of the transgenic seeds had germinated on the second day, while 84% of the wild-type seeds had germinated (Figure 3). On the fourth, fifth, and sixth days, the germination percentage of the seeds was more than 92% in both the wild-type and transgenic *A. thaliana* lines, with the exception of L4 (Figure 3). These data suggest that *LaSCL6* overexpression does not affect the meristem’s reactivation from dormancy.

### 2.4. LaSCL6 Overexpression Prolongs Juvenile Period in A. thaliana

**Figure 4:**
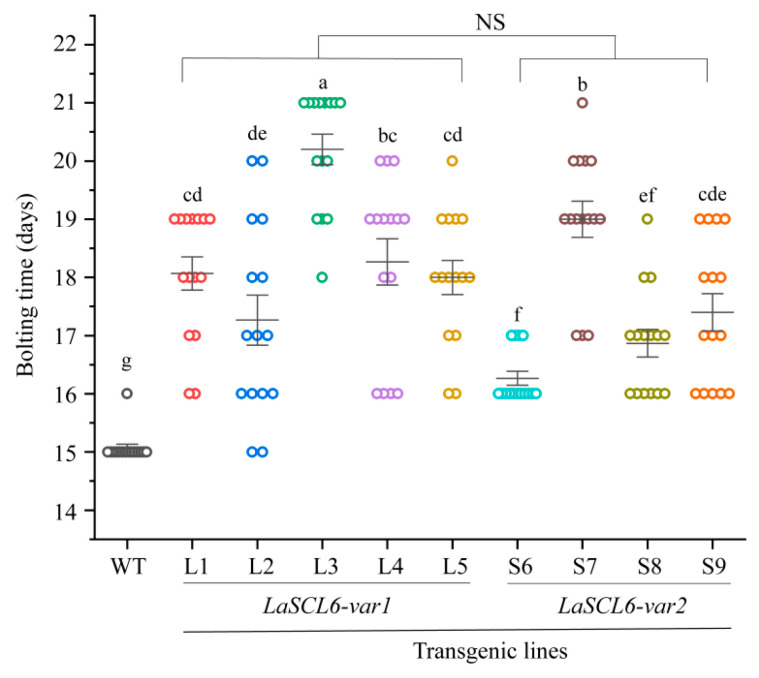
Bolting times of wild-type (WT) and transgenic *Arabidopsis thaliana*. The differences between each line were analyzed using LSD, *p* ≤ 0.05, indicated by lowercase letters. Student’s *t*-tests were used to compare *LaSCL6-var1* and *LaSCL6-var2* groups, *p* ≤ 0.05, and NS indicating no significant difference.

After determining the bolting time of *A. thaliana*, we found that it was delayed by *LaSCL6* overexpression. For the wild type, ~15 days were needed to bolt after being transferred into the soil, while, for the transgenic *A. thaliana*, 15–21 days were needed (mean ± SD, 17.93 ± 1.60) (Figure 4). In addition, there was no difference in bolting time between the two types of transgenic *A. thaliana*. For the *LaSCL6-var1*-overexpressing *A. thaliana*, 15–21 days (mean ± SD, 18.36 ± 1.62) were needed to bolt; for the *LaSCL6-var2*-overexpressing *A. thaliana*, 16–21 days (mean ± SD, 17.38 ± 1.42) were needed. In conclusion, the transgenic *A. thaliana* bolted later than the wild-type *A. thaliana*, indicating that the transition from the vegetative meristem to inflorescence was delayed by *LaSCL6* overexpression.

**Figure 5:**
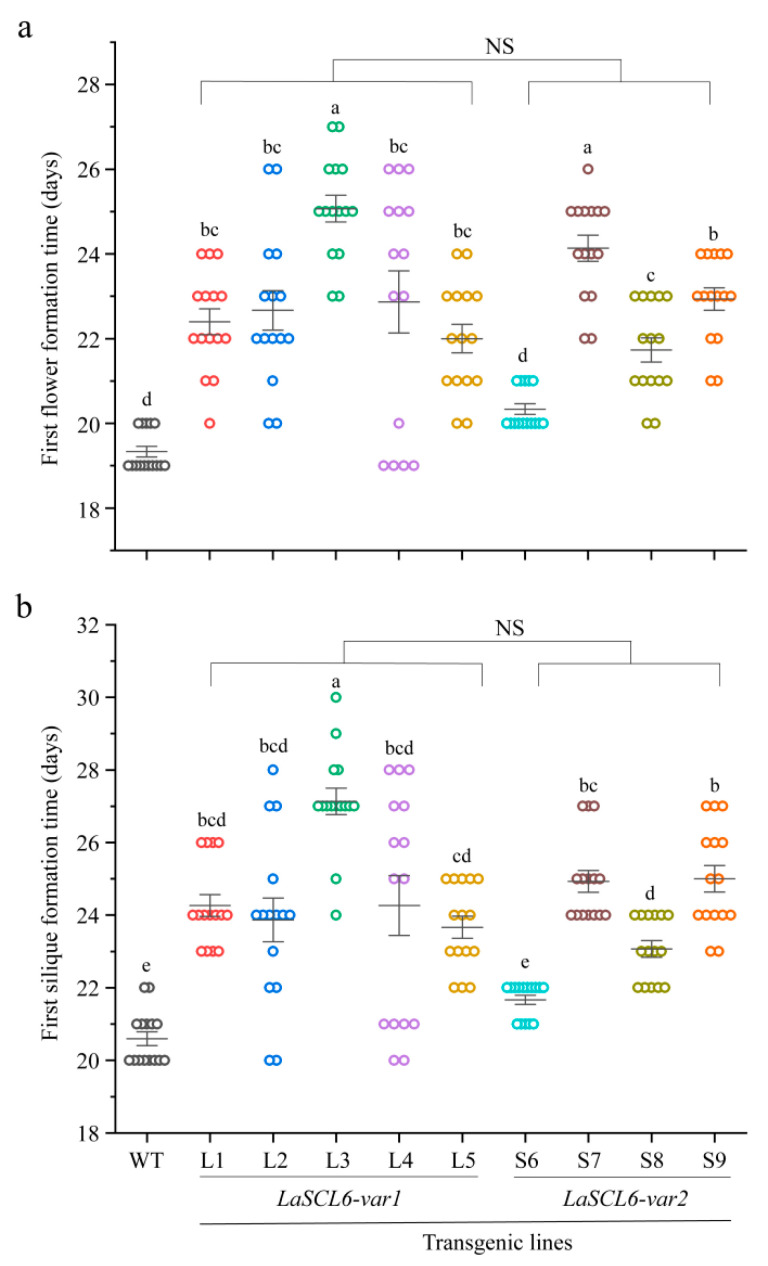
Formation times for the first flower (**a**) and silique (**b**) in wild-type (WT) and transgenic *Arabidopsis thaliana*. The differences between each line were analyzed using LSD, *p* ≤ 0.05, indicated by lowercase letters. Student’s *t*-tests were used to compare *LaSCL6-var1* and *LaSCL6-var2* groups, *p* ≤ 0.05, and NS indicating no significant difference.

The transgenic *A. thaliana* started flowering later than the wild-type *A. thaliana*, because the transgenic *A. thaliana* required 19–27 days (mean ± SD, 22.68 ± 1.93) to produce the first flower, while the wild-type *A. thaliana* required ~19 days (Figure 5a). The transgenic *A. thaliana* started fruiting later than the wild-type *A. thaliana* because the transgenic *A. thaliana* required 20–30 days (mean ± SD, 24.21 ± 2.14) to produce the first silique, while the wild-type *A. thaliana* required ~21 days (Figure 5b). However, there was no difference between the two types of transgenic *A. thaliana*. In conclusion, the transgenic *A. thaliana* started flowering later than the wild-type *A. thaliana*, indicating that the transition from the inflorescence meristem to the flower meristem was delayed by *LaSCL6* overexpression.

### 2.5. LaSCL6 Overexpression Delays Global Proliferative Arrest (GPA) in A. thaliana

**Figure 6:**
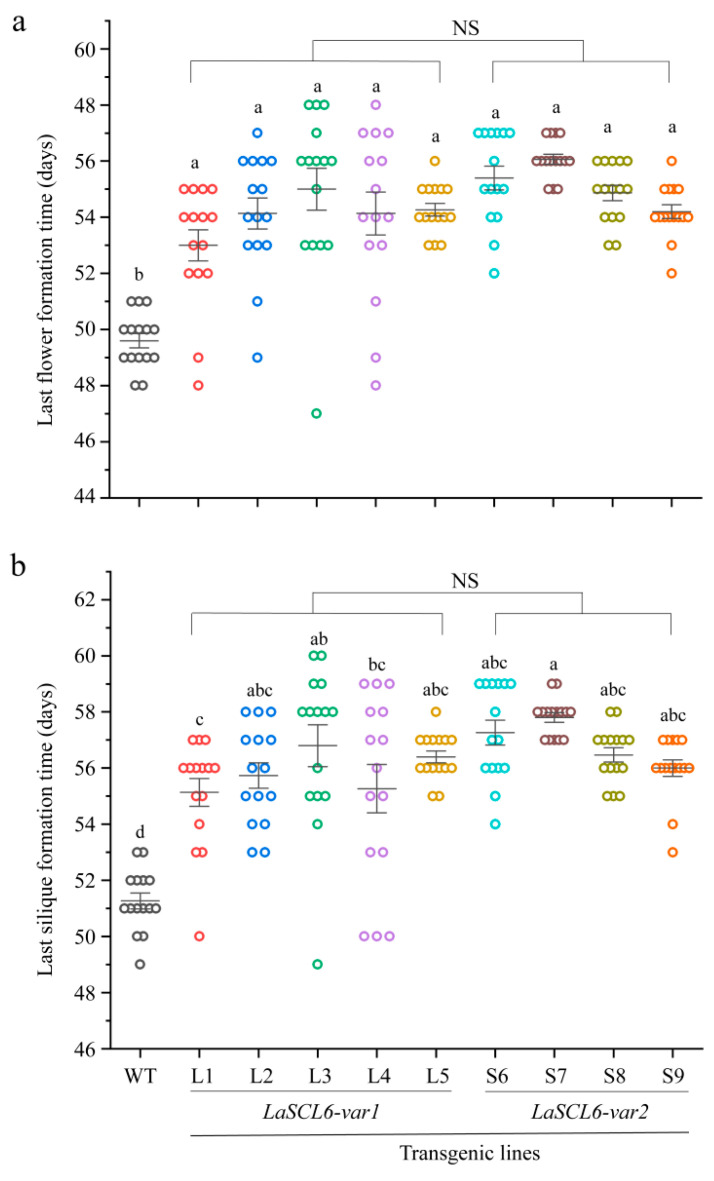
Formation times for the last flower (**a**) and silique (**b**) in wild-type (WT) and transgenic *Arabidopsis thaliana*. The differences between each line were analyzed using LSD, *p* ≤ 0.05, indicated by lowercase letters. Student’s *t*-tests were used to compare *LaSCL6-var1* and *LaSCL6-var2* groups, *p* ≤ 0.05, and NS indicating no significant difference.

After calculating the formation time for the last flower of *A. thaliana*, we found that the last flower formed later in the transgenic *A. thaliana* than the wild-type *A. thaliana*, because the transgenic *A. thaliana* required 47–58 days (mean ± SD, 54.44 ± 1.88) to produce the last flower, while the wild-type *A. thaliana* required ~50 days (Figure 6a). We counted the number of siliques each day until it became stable. We found that it stopped increasing in the transgenic *A. thaliana* at 49–60 days (mean ± SD, 56.13 ± 2.01) and in the wild-type *A. thaliana* at ~51 days (Figure 6b). However, there was no difference between the two types of transgenic *A. thaliana*. These data indicate that GPA occurred later in the transgenic *A. thaliana*, and it was delayed by *LaSCL6* overexpression. In addition, there was almost no difference in the timing of the formation of the last flower or silique between the two types of transgenic *A. thaliana*.

### 2.6. LaSCL6 Overexpression Increases Inflorescence Axis Length of A. thaliana

**Figure 7:**
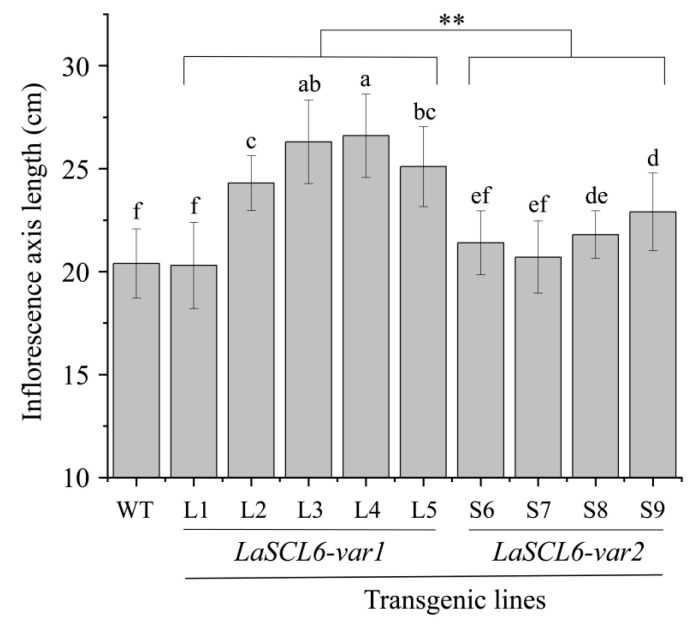
Statistical diagram of inflorescence axis lengths of wild-type (WT) and transgenic *Arabidopsis thaliana*. The differences between each line were analyzed using LSD, *p* ≤ 0.05, indicated by lowercase letters. Student’s *t*-tests were used to compare *LaSCL6-var1* and *LaSCL6-var2* groups, *p* ≤ 0.01, and ** was used.

After measuring the length of the inflorescence axis of *A. thaliana*, we found that *LaSCL6* overexpression promoted the elongation of the inflorescence axis because it was longer in the transgenic *A. thaliana* than in the wild-type *A. thaliana* (Figure 7). It was 17–29 cm (mean ± SD, 24.50 ± 2.92) and 19–26 cm (mean ± SD, 21.70 ± 1.75) in the *LaSCL6-var1*- and *LaSCL6-var2*-overexpressing *A. thaliana*, respectively, while it was 18–22 cm (mean ± SD, 20.40 ± 1.68) in the wild-type *A. thaliana* (Figure 7). In addition, it was longer in the *LaSCL6-var1*-overexpressing *A. thaliana* than in the *LaSCL6-var2*-overexpressing *A. thaliana* (Figure 7). In conclusion, the transgenic *A. thaliana* had a longer inflorescence axis.

**Figure 8:**
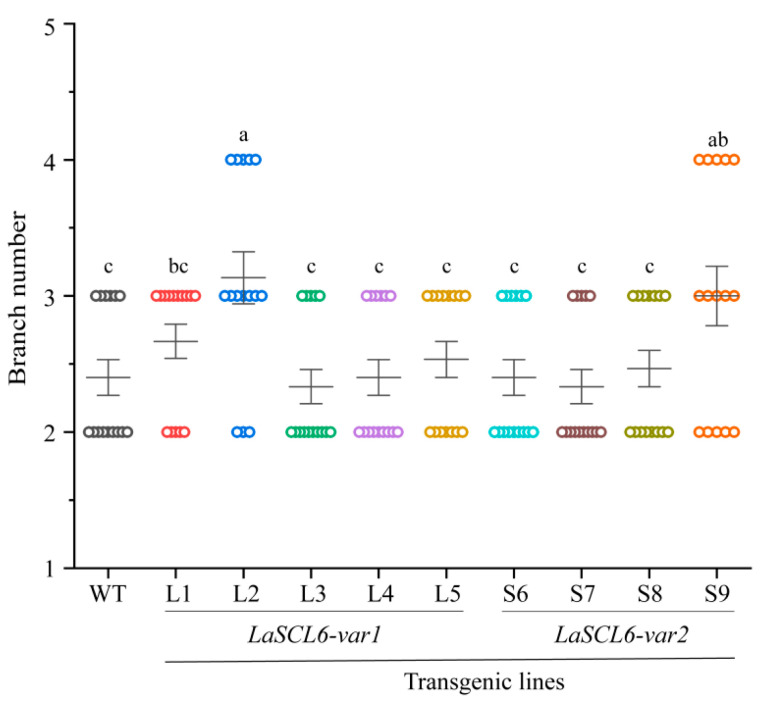
Branch numbers of wild-type (WT) and transgenic *Arabidopsis thaliana*. The differences between each line were analyzed using LSD, *p* ≤ 0.05, indicated by lowercase letters.

Regarding the branch number in *A. thaliana*, we found that it was two to three in both the wild-type and transgenic *A. thaliana*, with the exception of some L2 plants (Figure 8), indicating that *LaSCL6* overexpression had almost no influence on the occurrence and activity of the axillary meristem.

### 2.7. LaSCL6 Overexpression Influences Silique Yield of A. thaliana

**Figure 9:**
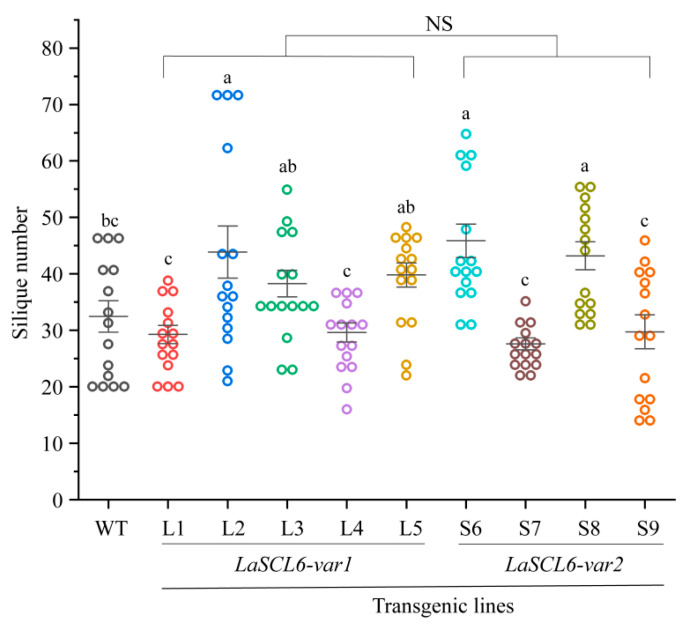
The number of siliques in wild-type (WT) and transgenic *Arabidopsis thaliana*. The differences between each line were analyzed using LSD, *p* ≤ 0.05, indicated by lowercase letters. Student’s *t*-tests were used to compare *LaSCL6-var1* and *LaSCL6-var2* groups, *p* ≤ 0.05, and NS indicating no significant difference.

After determining the fruit number of *A. thaliana*, we found that *LaSCL6* overexpression had varying influences on fruit production in each transgenic line. It was ~32 in the wild-type *A. thaliana*, while it was 16–73 (mean ± SD, 36.16 ± 11.78) and 14–66 (mean ± SD, 36.60 ± 12.44) in the *LaSCL6-var1*- and *LaSCL6-var2*-overexpressing *A. thaliana*, respectively (Figure 9). However, there was no difference between the two types of transgenic *A. thaliana*. These results indicate that *LaSCL6* overexpression affected the silique yield of *A. thaliana*.

## 3. Discussion

The meristem is an indispensable part of a plant that determines its morphology and function. The root meristem can continuously divide to produce new root cells, thereby promoting the growth of the roots. Many studies have shown that *SCL6* regulates the growth of roots [7,12,18]. Transgenic *A. thaliana* plants with miR171c overexpression and the *scl6-II scl6-III scl6-IV* triple mutant exhibited reduced root lengths [12]. The root lengths of *SCL6*-overexpressing and STTM171-silenced plants become longer, and the number of roots increased, while the root length of miR171-overexpressing *Lilium pumilum* DC. Fisch became shorter and the number of roots decreased [18]. However, the root length of *SlGRAS24*-overexpressing *Solanum lycopersicum* L. cv. Micro-Tom was obviously shortened [20]. In this study, the root lengths of two types of *LaSCL6*-overexpressing *A. thaliana* were shorter, showing that *LaSCL6* has a similar role to *SlGRAS24*. These data indicate that *SCL6* plays a role in the growth of plant roots via different regulatory mechanisms, and further research is needed to explore these differences.

Previous studies have shown that the miR171-*SCL6* module affects the plant height [17,20,21]. For example, miR171b-overexpressing rice was taller [21], which was consistent with the results obtained for *S. lycopersicum* [20]. Meanwhile, the height of barley became shorter after miRNA171 overexpression [17]. In this study, *LaSCL6* overexpression promoted inflorescence axis elongation, which was consistent with the result obtained for barley. In addition, the inflorescence axis was longer in *LaSCL6-var1*-overexpressing *A. thaliana* than in *LaSCL6-var2*-overexpressing *A. thaliana.*

The miR171-*SCL6* module affects shoot branching [7,12,15,30]. For example, miR171a- or miR171c-overexpressing plants had a reduced shoot branch number [12,15]. In *scl6-II scl6-III scl6-IV* triple mutant *A. thaliana*, there was a significant decrease in the branch number. In addition, *Petunia HAM* mutant plants had no lateral organs, and a ring mark structure was formed in the missing organs [7]. These studies indicate that *SCL6* plays a positive role in the regulation of shoot branching by controlling the meristem’s activity in the lateral bud. However, in this study, there was almost no change in the branch number of *A. thaliana* after *LaSCL6* overexpression (Figure 8). Notably, after *LaSCL6-var2* (*LkHAM*) was overexpressed in the *A. thaliana ham123* mutant, normal branches were able to initiate from the cauline leaves of transgenic *A. thaliana* [31]. These data add complexity to the study of the functional mechanism of *LaSCL6* in shoot branching.

**Figure 10:**
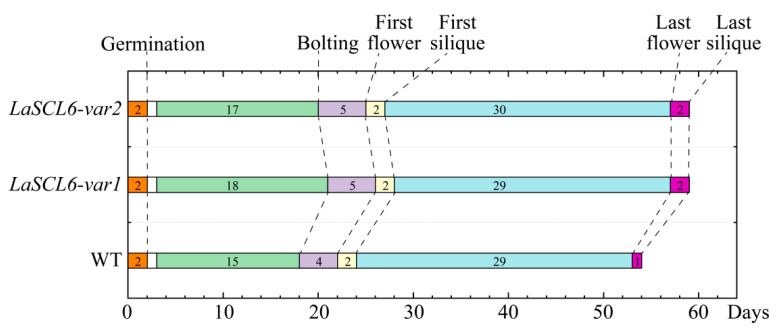
Occurrence of life cycle events in wild-type and transgenic *Arabidopsis thaliana.* When *A. thaliana* seeds were transported to a growth chamber for two days, the germination percentage was more than 78%; when the seedlings had 2–3 true leaves, the plants were transferred into the soil, and the timings of life cycle events including bolting and flower and silique formation were recorded. The white boxes indicate the number of days before the seedlings were transferred into the soil. The numbers in the boxes indicate the duration of each stage.

The miR171-*SCL6* module also has an effect on the timing of the plant phase transition [17,20]. The first flower of Sly-miR171-overexpressing plants formed late [20]. In barley, miR171 overexpression altered the vegetative to reproductive phase transition by activating the miR156 pathway and repressing the expression of the *THIRD OUTER GLUME* and *Hordeum vulgare* L. cv. Golden promise *Plastochron1* genes [17]. In this study, the bolting time and the formation time of the flower (the first and the last) and silique (the first and the last) of *LaSCL6*-overexpressing *A. thaliana* were later than those of the wild-type *A. thaliana* (Figure 4, Figure 5 and Figure 6). After determining the timing of these life cycle events in *A. thaliana*, we found that the juvenile period in *LaSCL6*-overexpressing plants was longer, and the bolting time in *LaSCL6-var1* and *LaSCL6-var2* was 3 and 2 days later than that in the wild-type *A. thaliana*, respectively (Figure 10). The time from bolting to the first flower formation was 1 day later in the *LaSCL6*-overexpressing *A. thaliana* than in the wild-type *A. thaliana*, but there was no difference between the two types of transgenic plants (Figure 10). Overall, the life cycle in transgenic plants was prolonged by approximately 5 days. These data show that *LaSCL6* overexpression inhibited the transitions from the vegetative meristem to inflorescence meristem and from the flower meristem to meristem arrest in *A. thaliana*.

## 4. Materials and Methods

### 4.1. Plant Materials and Growth Conditions

The seeds of *A. thaliana* ecotype Columbia (Col-0), stored in our laboratory, were disinfected in a 0.8% NaClO solution and then inoculated on 1/2 Murashige and Skoog medium at 4 °C for 3 days. Then, the seeds were transported to a growth chamber with a 16 h photoperiod, a temperature of 22 °C, and relative humidity of 75–85%. When the seedlings had 2–3 true leaves, twenty plants of each line were transferred into 1:1 mixed roseate and nutrient soil and some were sampled for genomic DNA and total RNA extraction. After sampling, the materials were immediately frozen in liquid nitrogen and then stored at −80 °C.

### 4.2. Plasmid Construction and Genetic Transformation

The primers were designed based on our published *LaSCL6-var1* (GenBank: MK501379) and *LaSCL6-var2* (GenBank: JX280920) mRNA sequences. After the Noc I restriction site was added to the forward primers and the PmL I restriction site was added to the reverse primers, the primers 5′-ACGGGGGACTCTTGACCATGGGGATGAACGGGATGCTAAGCAGG-3′ and 5′-CTGGTCACCAATTCACACGTGTTAAGGCGGGGGCCCGCACCT-3′ were used to clone *LaSCL6-var1* and the primers 5′-ACGGGGGACTCTTGACCATGGGGATGGAAGATTTGGAGAGTATG-3′ and 5′-CTGGTCACCAATTCACACGTGTTAAGGCGGGGGCCCGCACCT-3′ were used to clone *LaSCL6-var2* into the binary vector pCAMBIA1305.1. Then, these vectors were used to transform *A. thaliana* ecotype Col-0 using the floral dip method, mediated by the *Agrobacterium* tumefaciens strain GV3101. The homozygous T3 transgenic plants, which were cultured in the same way as the wild-type *A. thaliana*, were used for the phenotype investigation.

### 4.3. Extraction of Nucleic Acids, PCR, and qRT-PCR

Genomic DNA was extracted from *A. thaliana* using the Plant Genomic DNA Extraction Kit (BioTeke, Beijing, China), following the manufacturer’s protocol. The quality of the DNA was determined using a spectrophotometer and agarose gel electrophoresis. Then, the DNA was used for PCR with the specific primers of *LaSCL6*: 5′-TCCCACATTGTCTAACCAGCC-3′ and 5′-GCGGGATTCGAACCGTAGAC-3′.

The total RNA was extracted from *A. thaliana* using the Easy Pure RNA Kit (TRANS; Beijing, China), following the manufacturer’s protocol. Then, 2 µg of total RNA was reverse-transcribed into cDNA with the TransScript II One-Step gDNA Removal and cDNA Synthesis SuperMix (TRANS; Beijing, China). The qRT-PCR was performed with the Bio-Rad CFX96 PCR system using TB Green^®^ Premix Ex Taq™ (Tli RNase H Plus) (Takara; Shiga, Japan). *AtUBQ1* (AT3G52590) was used as the internal control with the specific primers 5′-GCCAAGATCCAAGACAAAGAAG-3′ and 5′-CTGATTGTACTTACGAGCAAGC-3′ [32]. The relative gene expression levels were calculated using the 2^−∆∆Ct^ method. The qRT-PCR was performed with three replicates, and the data are presented as the mean ± SD.

### 4.4. Phenotypic Observation and Statistical Analysis

Fifty seeds were used for germination percentage measurement after they were transferred to the incubator. Twenty seedlings were randomly selected for root length measurement after they were transferred to the incubator for 8 days.

Fifteen seedlings in each line were randomly selected from twenty seedlings planted in the soil to measure the bolting time, the flower and silique formation times, the length of the inflorescence axis, and the number of branches and siliques.

Excel was used for data statistics and analysis, and Origin was used for drawing. The significance of the differences between wild-type and transgenic *A. thaliana* was analyzed with Statistical Product and Service Solutions (SPSS Statistics 26, IBM Corp., New York, NY, USA) software using analysis of variance (ANOVA). Student’s *t*-tests were used to compare *LaSCL6-var1* and *LaSCL6-var2* groups.

## 5. Conclusions

Taken together, our results show that *LaSCL6* plays a role in the transition and maintenance of the meristem, as well as the growth of roots and the plant height, providing more functional information about *LaSCL6* with respect to the whole life cycle.

## Data Availability

The data presented in this study are available upon reasonable request from the corresponding author.
